# Bivalent binding of staphylococcal superantigens to the TCR and CD28 triggers inflammatory signals independently of antigen presenting cells

**DOI:** 10.3389/fimmu.2023.1170821

**Published:** 2023-05-03

**Authors:** Martina Kunkl, Carola Amormino, Francesco Spallotta, Silvana Caristi, Maria Teresa Fiorillo, Alessandro Paiardini, Raymond Kaempfer, Loretta Tuosto

**Affiliations:** ^1^ Department of Biology and Biotechnologies “Charles Darwin”, Sapienza University, Rome, Italy; ^2^ Laboratory affiliated to Istituto Pasteur Italia-Fondazione Cenci Bolognetti, Sapienza University, Rome, Italy; ^3^ Department of Biochemical Sciences “A. Rossi Fanelli”, Sapienza University of Rome, Rome, Italy; ^4^ Department of Biochemistry and Molecular Biology, The Institute for Medical Research Israel-Canada, The Hebrew University-Hadassah Medical School, Jerusalem, Israel

**Keywords:** staphylococcal superantigens, T cells, TCR (T cell receptor), CD28, inflammation

## Abstract

*Staphylococcus aureu*s superantigens (SAgs) such as staphylococcal enterotoxin A (SEA) and B (SEB) are potent toxins stimulating T cells to produce high levels of inflammatory cytokines, thus causing toxic shock and sepsis. Here we used a recently released artificial intelligence-based algorithm to better elucidate the interaction between staphylococcal SAgs and their ligands on T cells, the TCR and CD28. The obtained computational models together with functional data show that SEB and SEA are able to bind to the TCR and CD28 stimulating T cells to activate inflammatory signals independently of MHC class II- and B7-expressing antigen presenting cells. These data reveal a novel mode of action of staphylococcal SAgs. By binding to the TCR and CD28 in a bivalent way, staphylococcal SAgs trigger both the early and late signalling events, which lead to massive inflammatory cytokine secretion.

## Introduction

*Staphylococcus aureus* is a gram-positive bacterium, which persists as commensal in the human population. It can cause serious infections leading to toxic shock syndrome (TSS) and sepsis, with an overall mortality rate of 25% (1–3). Type I toxins secreted by *Staphylococcus aureus* are the most important virulence factors. They, indeed, act as superantigens (SAgs) and stimulate T cells to produce massive inflammatory cytokines, thus leading to vascular damage, multi-organ system breakdown and fatal shock (4–6). By binding MHC class II and B7 molecules on antigen presenting cells (APCs) as well as specific variable regions within the TCR β chain (TCRVβ) and the costimulatory receptor CD28, staphylococcal SAgs activate polyclonally a large proportion of T cells, which in turn produce high levels of inflammatory cytokines (4, 7).

Staphylococcal enterotoxin A (SEA) and B (SEB) were the first SAgs to be identified (8, 9) and their three-dimensional structures were solved by crystallography (10, 11), followed by other staphylococcal SAg structures (12). They share a compact two-domain fold with a β-barrel (oligonucleotide OB-fold) at the N-terminal domain and a β-grasp fold at the C-terminal domain. Both SEB and SEA use hydrophobic residues at the N-terminal OB-domain to bind the α-chain of MHC class II molecules on APCs, outside the MHC class II peptide-binding groove (13, 14). Furthermore, SEA binds the β-chain of MHC class II on the opposite side through a tetravalent zinc bridge at the C-terminal domain (15). The interaction of both SEB and SEA with particular elements within the TCRVβ, mainly the complementary-determining region 2 (CDR2) and, to a lesser extent, the hypervariable region 4 (HV4), is mediated by distinct residues located in a shallow groove between the N-terminal β-barrel and the α (2)-helix (16–18). Thus, staphylococcal SAgs engage TCRVβ and MHC class II molecules through distinct regions enabling the formation of a ternary complex, which transmits TCR-dependent signals (19).

To elicit massive inflammatory cytokine production, staphylococcal SAgs also bind and stimulate the costimulatory receptor CD28 and its coligands B7.1/CD80 and B7.2/CD86 (20–23) through a highly conserved 12 amino-acid β-strand (8)/hinge/α-helix (24) domain (25). This domain is distal from the TCR and MHC class II binding sites and engages the homodimer interfaces of CD28 and B7, thus enhancing the CD28/B7 interaction also in the absence of MHC class II (20–23, 26). Moreover, SEB binding into the CD28/B7 costimulatory axis also favours TCR recruitment into the immunological synapse and inflammatory signals in a MHC class II independent manner (27).

Here we explored the capability of staphylococcal SAgs to interact with the TCR and CD28 molecule and to trigger inflammatory signals in the absence of APCs. Indeed, our computational modelling suggested that both SEB and SEA may bind to the TCR and CD28 by using distinct regions on the same side facing the membrane bilayer, even in the absence of MHC class II and B7 molecules. Consistently with our computational simulations, the bivalent binding of both SEB and SEA to the TCR and CD28 triggered both early and late signalling events leading to inflammatory cytokine production in human primary CD4^+^ T cells. Finally, our data on both the colocalization of the TCR and CD28 in SEB/SEA-stimulated cells demonstrate that, by bridging simultaneously the TCR and CD28, staphylococcal SAgs favour the cooperative activation of inflammatory signals even in the absence of MHC class II- and/or B7-expressing APCs.

## Materials and methods

### Structural modelling of the interaction between SEA, SEB, TCR and CD28

Structure predictions of full-length proteins were performed in a standalone version of AlphaFold2 and Alpha Fold-Multimer (28), as implemented in ColabFold, which was run on a local computer with Ubuntu 22 operating system and accelerated with two NVIDIA GeForce RTX 2080 Ti GPU. “Template mode” using the following experimentally determined structures was used to this purpose: SEA (PDB: 1ESF) (11); SEB (PDB: 1SEB) (17); CD28 extracellular domain (PDB: 1YJD) (29); CD28 transmembrane helices (PDB: 7VU5) (30); TCR (TRAV22/TRBV19) – MHC class II (HLA-DR1), (PDB:4C56) (19); B7.2/CD86 (PDB:1I85) (31); TCR in association with the CD3γϵ-CD3δϵ-CD3ζζ signalling hexamer (PDB: 6JXR) (32). Protein-Protein Docking was done with ClusPro 2.0 with Immunoglobulin (Ig)-like structures docking (33), HADDOCK (34), using as input for spatial restraints the previously obtained structures of SEA with the TCR (TRAV22/TRBV7-9, PDB: 5FK9) (18), SEB with the TCR (PDB:4C56) (19) and mutational analysis carried out by Kaempfer et al. for SEB and CD28 (21). Standalone MultiLZerD was used for multiprotein docking (35). Other parameters were kept at their default values. Protein sequence manipulations, superpositions and modelling were carried out using PyMod 3.0 (36). Root-mean-square deviation (RMSD) values between structures were calculated using PyMod 3.0.

### Cells, antibodies and reagents

Human primary CD4^+^ T cells were isolated from peripheral blood mononuclear cells (PBMC) by negative selection using a EasySepTM isolation kit (#17952, STEMCELL Technology, CAN) and cultured in RPMI 1640 supplemented with 5% human serum (Euroclone, UK), L-glutamine, penicillin and streptomycin. The purity of the sorted population was > 95%, as evidenced by staining with anti-CD3 plus anti-CD4 Abs. PBMCs were derived from buffy coats of anonymous healthy blood (HD) donors provided by the Policlinico Umberto I (Sapienza University of Rome, Italy). Written informed consent was obtained from blood donors and both the informed consent form and procedure were approved by the Ethics Committee of Policlinico Umberto I (ethical code N. 1061bis/2019, 13/09/2019).

CD28-negative Jurkat T cell line CH7C17 (37), CH7C17 cells, stably transfected with human CD28WT (38) and the TCR-negative 31.13 Jurkat T cell line (39) were maintained in culture as previously described (40, 41). Murine L cells (5-3.1/B7) co-transfected with HLA-DRB1*0101 and B7.1/CD80 were used as APCs (27).

The following antibodies were used: anti-human CD86-PE (#560957), anti-human CD28-PE (#561793) (BD Biosciences, Italy); anti-human CD80-FITC (#21270803), anti-human CD4-APC (#21850046), anti-human CD3-PE (#21620034), anti-HLA-DR-PE (#21819984) (ImmunoTools, Germany); rabbit anti-human phosphorylated Y319 ZAP-70 (#2701), rabbit anti-human NF-κB/p65 (#8242) (Cell Signalling Technologies, USA); rabbit anti-human phosphorylated Y783 PLC-γ1 (#sc-12943) (Santa Cruz Biotechnology, USA); mouse anti-human CD3 (OKT3, ECACC 86022706); goat anti-mouse Alexa-flour 594 (#A11020), goat anti-rabbit Alexa-flour 594 (#A11072), goat anti-mouse Alexa-flour 488 (#A11070), goat anti-rabbit Alexa-flour 488 (#A11070) (ThermoFisher Scientific, Italy). Staphylococcal Enterotoxin A (SEA, #59399) and Staphylococcal Enterotoxin B (SEB, #54881) were purchased by Merck (Italy).

### Cytokine production

Secretion of IFN-γ, IL-2, IL-6, TNF-α, IL-17A, IL-22 and GM-CSF was measured in the supernatants of CD4^+^ T cells cultured for the indicated times in flat-bottom 24-culture wells (2 x 10^6^ cells per well) either unstimulated or stimulated with 1 μg ml^-1^ SEB or 0.1 μg ml^-1^ SEA by using human IFN-γ (DY285), IL-2 (DY-202), IL-6 (DY-206), TNF-α (DY-210), IL-17A (DY-317), IL-22 (DY-782) and GM-CSF (DY-215) ELISA kits (Bio-Techne/R&D Systems, USA). Data were analysed by a Bio-Plex (Bio-Rad, Hercules, CA, USA). The assays were performed in duplicate. The sensitivity of the assay was 9.4 pg ml^-1^ for IL-6 and IFN-γ, 15.6 pg ml^-1^ for IL-2, TNF-α, IL-17A and GM-CSF, and 31.2 pg ml^-1^ for IL-22.

### Microscopy analysis of SEB and SEA binding, PLC-γ and ZAP-phosphorylation, and RelA/NF-κB nuclear translocation

SEB and SEA were labelled with Alexa-fluor-594 protein labelling kit, according to the manufacturer’s instructions (ThermoFisher Scientific). Primary CD4^+^ T cells or CH7 or CD28WT or 31.13 Jurkat cells were incubated with 1 μg ml^-1^ SEB-Alexa-fluor-594 or 0.1 μg ml^-1^ SEA-Alexa-fluor-594 for different times. For the analysis of phosphorylation of PLC-γ1 in Y783 (pPLC-γ1) or ZAP-70 in Y319 (pZAP-70), primary CD4^+^ T cells were stimulated 1 μg ml^-1^ SEB or 0.1 μg ml^-1^ SEA for different times.

Following the cells were fixed by 3% paraformaldehyde and seeded on poly-L-lysin (#8920, Sigma) coated-cover glasses (12 mm). The cells were washed in PBS and permeabilised by 0.1% saponin in PBS containing 1% BSA. pPLC-γ1 and pZAP-70 staining were performed by using anti-human pPLC-γ (1:100 dilution) and anti-human pZAP-70 (1:50 dilution) followed by goat anti-rabbit Alexa-flour 594 (1:150 dilution). Glasses were mounted onto slides with Vectashield^®^ mounting medium with 4′,6-diamidino-2-phenylindole dihydrochloride (DAPI) (H-1200; Vector Laboratories, Inc.; Burlingame, CA, USA). Images were obtained with a 63X oil objective, Zeiss Apotome fluorescence microscope, and Zen software (Zeiss, Oberkochen, Germany). The Mean Fluorescent Intensity (MFI) of SEB-594-Alexa-fluor-594 or SEA-Alexa-fluor-594 or pPLC-γ1 or pZap-70 were quantified by using Fiji ImageJ software. At least one hundred cells were examined quantitatively for each condition in three independent experiments.

RelA/NF-κB nuclear translocation in primary CD4^+^ T cells unstimulated or stimulated for the indicated times with 1 μg ml^-1^ SEB or 0.1 μg ml^-1^ SEA was performed by using anti-human RelA (1:300 dilution) followed by goat anti-rabbit Alexa-flour 488. Nuclei were labelled with DAPI. Images of RelA localization were obtained with a computer-controlled Nikon Eclipse 50i epifluorescence microscope and a plan achromat microscope objective 100XA/1.25Oil OFN22 WD 0.2 and QImaging QICAM Fast 1394 Digital Camera, 12-bit,Mono (Minato, Tokyo, Japan). The fluorescence intensities of cytoplasmic and nuclear RelA, overlapping with DAPI, were quantified by using Fiji ImageJ software and intensities ratio were calculated for each cell. At least seventy cells were examined quantitatively for each condition in three independent experiments.

### Confocal microscopy analysis of the colocalization of CD3 and CD28 with SEB or SEA

Primary CD4^+^ T cells incubated for 5 minutes with 1 μg ml^-1^ SEB-Alexa-fluor-594 or 0.1 μg ml^-1^ SEA-Alexa-fluor-594 were fixed by 3% paraformaldehyde and seeded on poly-L-lysin (#8920, Sigma) coated-cover glasses (12 mm). After washing and permeabilization with 0.1% saponin in PBS containing 1% BSA, CD28 and CD3 staining were performed by using rabbit anti-human CD28 (1:100 dilution) followed by goat anti-rabbit Alexa-flour 488, and mouse anti-human CD3 (OKT3) followed by goat anti-mouse Alexa-flour 488 (1:150 dilution). Nuclei were stained with DAPI. Confocal observations were performed with a 63X oil objective, Nikon Eclipse Ti2 confocal microscope, and Z stack images were processed by NIS Elements AR 5.30 software (Nikon Europe B.V.) using the same acquisition settings. The colocalizations of CD28 or CD3 with SEB or SEA were quantified by using Fiji ImageJ software. The Pearson correlation coefficient (PCC) in each cell was calculated with a range +1 (perfect correlation) to -1 (perfect exclusion) (42).

### Statistical analysis

The sample size was chosen based on previous studies to ensure adequate power. Parametrical statistical analysis (mean and standard deviation) was performed to evaluate differences between continuous variables through Prism 8.0 (GraphPad Software, San Diego, CA), using Student’s *t* test or one-way ANOVA with the Fisher’s LSD test for multiple comparisons. For all tests, P values < 0.05 were considered significant.

## Results

### SEB and SEA activate T cells and induced inflammatory cytokines independently of MHC-II and B7 molecules

**Figure 1:**
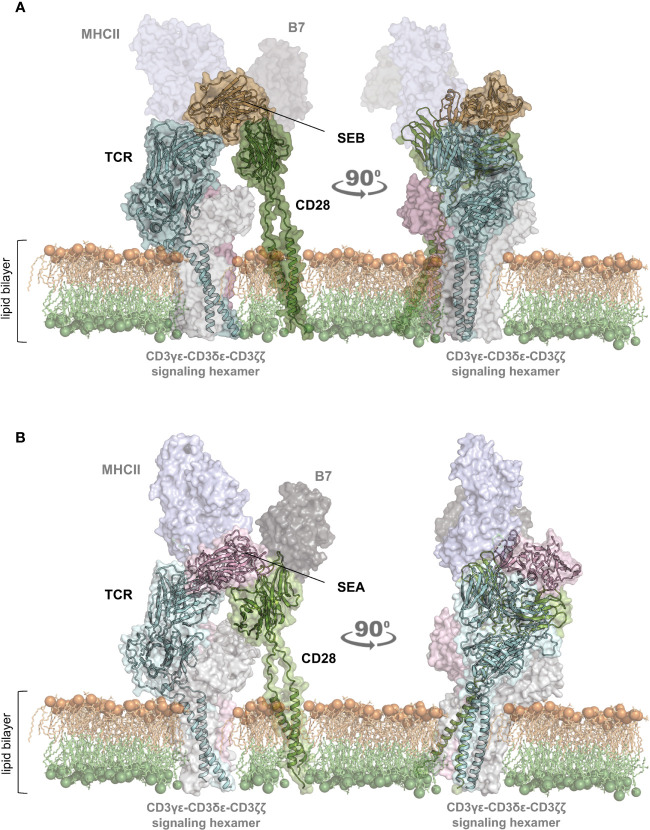
Structural model of the TCR–SEB/SEA–CD28 complex. **(A, B)** Cartoon representation with transparent surface of the overall structure of SEB (PDB: 1SEB) (17) in brown or SEA (PDB: 1ESF) (11) in violet in a complex with the TCR (TRAV22/TRBV19) in cyan **(A)**; PDB:4C56) (19) and TRAV22/TRBV7-9 **(B)**; PDB: 5FK9) (18) or CD28 (green; PDB: 1YJD, PDB: 7VU5) (29, 30)). The lipid bilayer is shown as reference. Shadowed surfaces of MHC class II (MHCII; PDB:4C56), B7 (PDB:1I85) (31), and the CD3γϵ-CD3δϵ-CD3ζζ signalling hexamers (PDB: 6JXR) (32) are also shown as reference.

To gain insights into the structural mechanism governing the interaction between staphylococcal SAgs and their full-length interactors, i.e. TCR/CD3γϵ-CD3δϵ-CD3ζζ hexamer, CD28, MHC class II and B7, we used computational modelling based on the recently released artificial intelligence (AI)-based AlphaFold2 algorithm (28), together with the experimental availability of the SEB binding domains of TCR/CD3γϵ-CD3δϵ-CD3ζζ, CD28, MHC class II (HLA-DR1) and B7.2/CD86 (**Figure 1A**; **Supplementary Movie S1**) (17, 19–21). Similarly, it was also possible to study the complex formed by SEA with TCR/CD3γϵ-CD3δϵ-CD3ζζ, CD28, MHC class II and B7.2/CD86 (**Figure 1B**; **Supplementary Movie S2**) (11, 15, 18, 21).

**Figure 2:**
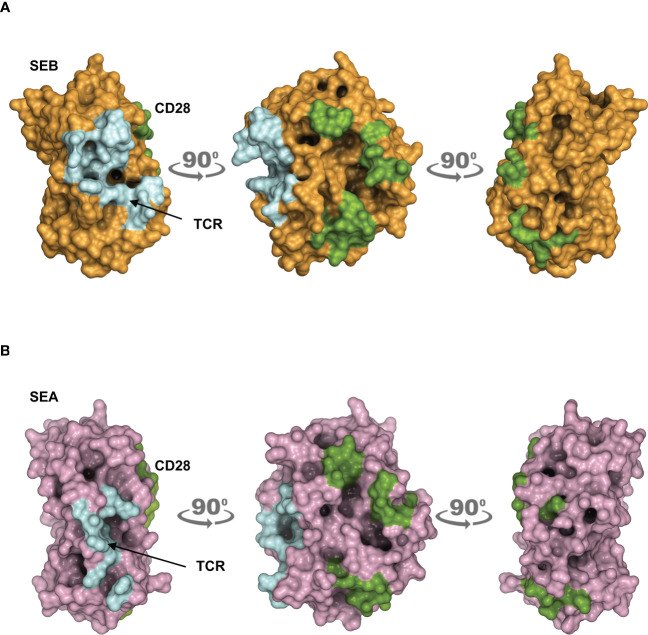
Contact maps on the surface area of SEB and SEA. **(A, B)** The structural model of the TCR–SEB/SEA–CD28 complex was used to obtain the extent of interaction between SEB/SEA and their ligands. Surface representation of SEB in brown **(A)** or SEA in violet **(B)** with the surface area of interaction (SAI) representing the contacts with the TCR (cyan) and CD28 (green). The following SAI values were measured: SEB/CD28 ≈ 1320 Å^2^, SEB/TCR ≈ 1380 Å^2^; SEA/CD28 ≈ 1580 Å^2^, SEA/TCR ≈ 1266 Å^2^.

The obtained models are in agreement with previous data showing the interaction of SEB with the TCR, CD28 and MHC class II molecules (21). CD28 is predicted to bind to a distant region of SEB (**Figure 1A**) or SEA (**Figure 1B**) compared to the TCR binding sites, but on the same side facing the membrane bilayer. In agreement with Arad et al. (20) and Levy et al. (23), SEB adopts a wedge-like conformation in binding both the TCR and CD28, with the residues 150–161 in the β-strand_8_/hinge/α-helix_4_ domain of SEB in close contact with the CD28 homodimer interface (**Figure 1A**; **Supplementary Movie S1**). A very similar (RMSD ≈ 2.0 Å) conformation and orientation is predicted to be adopted by SEA in TCR and CD28 binding (**Figure 1B**; **Supplementary Movie S2**), as also evidenced by the co-crystallized structure of SEA with the TCR (**Supplementary Movie S2**) (18). Indeed, as shown by contacts mapping on the surface of SEB (**Figure 2A**), most of the accessible surface area of SEB is engaged in contacts of roughly the same extent with CD28 (≈ 1320 Å^2^) and the TCR (≈ 1380 Å^2^) and, to a lesser extent, with MHC class II (≈ 950 Å^2^) and B7 (≈ 500 Å^2^). Given the very similar interaction binding mode of SEA, the values of SEA contact area (**Figure 2B**) are in good agreement with those measured for SEB (TCR ≈1266 Å^2^; CD28 ≈1580 Å^2^). Therefore, the structural modelling of SAgs and their full-length interactors and the analysis of the interacting regions suggest that SEB and SEA might bind simultaneously to the TCR and CD28 even in the absence of MHC class II and B7 molecules, thus triggering inflammatory signals.

**Figure 3:**
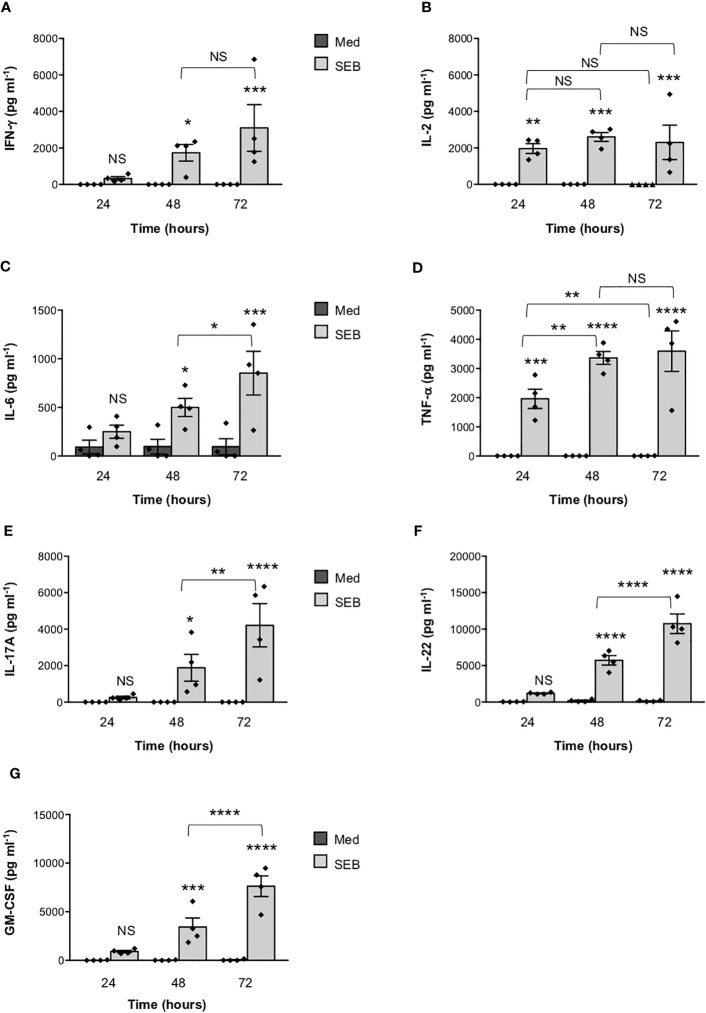
Stimulation of CD4+ T cells by SEB induces the secretion of inflammatory cytokines in the absence of MHC class II- and B7-expressing APCs. **(A–G)** Human CD4+ T cells isolated by the peripheral blood of healthy donors (HD) were unstimulated (Med) or stimulated for different times with 1 µg ml-1 SEB. IFN-γ **(A)**, IL-2 **(B)**, IL-6 **(C)**, TNF-a **(D)**, IL-17A **(E)**, IL-22 **(F)** and GM-CSF **(G)** levels in culture supernatant were measured by ELISA. Data show the mean ± SEM of different HD (n = 4). Statistical significance was calculated by One-way ANOVA. Means values (pg ml-1): 24 hours; IFN-γ, Med = 0, SEB = 328.7; IL-2, Med = 0, SEB = 1966; IL-6, Med = 92.2; SEB = 249; TNF-α, Med = 0, SEB = 1959; IL-17A, Med = 0, SEB = 251.7; IL-22, Med = 37.4, SEB = 1197; GM-CSF, Med = 19.1, SEB = 916.5. 48 hours; IFN-γ, Med = 0, SEB = 1738; IL-2, Med = 0, SEB = 2600; IL-6, Med = 96.7, SEB = 498.8; TNF-α, Med = 0, SEB = 3361; IL-17A, Med = 0, SEB = 1884; IL-22, Med = 178.6, SEB = 5726; GM-CSF, Med = 22,3, SEB = 3426. 72 hours; IFN-γ, Med = 0, SEB = 3099; IL-2, Med = 0.9, SEB = 2303; IL-6, Med = 95, SEB = 851.5; TNF-α, Med = 2.6, SEB = 3591; IL-17A, Med = 0, SEB = 4211; IL-22, Med = 140.9, SEB = 10737; GM-CSF, Med = 42.3, SEB = 7633. (*) p < 0.05, (**) p < 0.01, (***) p < 0.001, (****) p < 0.0001. NS, not significant.

**Figure 4:**
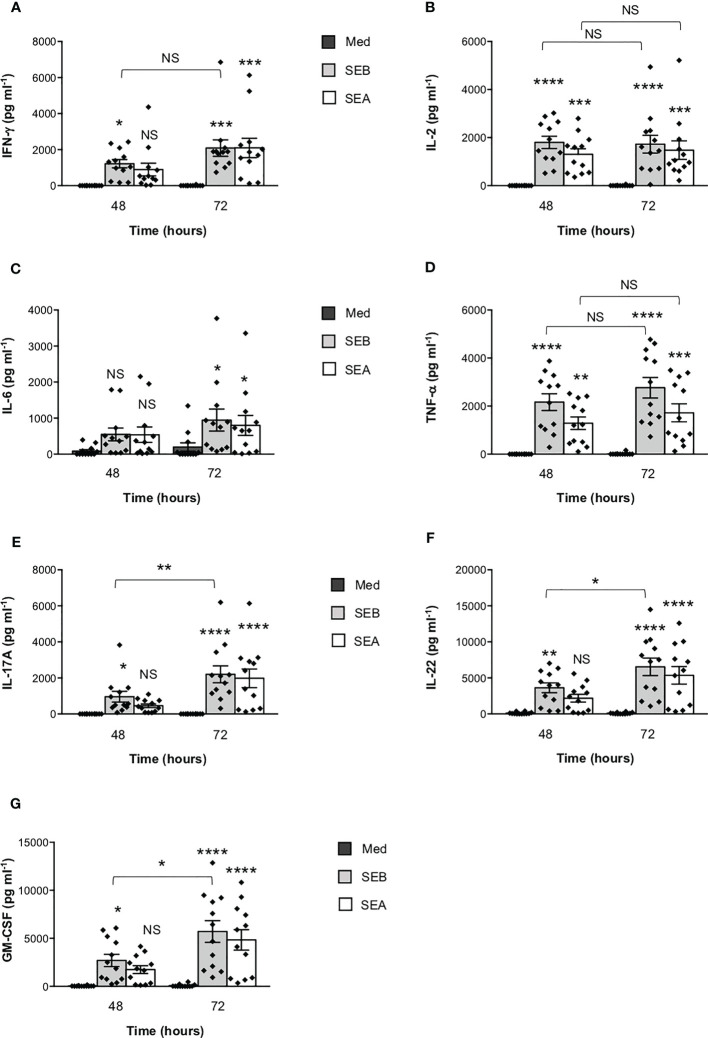
SEB and SEA induce comparable levels of inflammatory cytokines in CD4^+^ T cells. **(A–G)** Peripheral blood CD4^+^ T cells from HD (n = 12) were unstimulated (Med) or stimulated with SEB (1 μg ml^-1^) or SEA (0.1 μg ml^-1^) for the indicated times. IFN-γ **(A)**, IL-2 **(B)**, IL-6 **(C)**, TNF-α **(D)**, IL-17A **(E)**, IL-22 **(F)** and GM-CSF **(G)** levels in culture supernatant were measured by ELISA. Data show the mean ± SEM and statistical significance was calculated by one-way ANOVA. Means values (pg ml^-1^): **48 hours**; IFN-γ, Med = 0.5, SEB = 1219, SEA = 897.1; IL-2, Med = 0.7, SEB = 1804, SEA = 1305; IL-6, Med = 85.9, SEB = 549.1, SEA = 539.1; TNF-α, Med = 0, SEB = 2166, SEA = 1286; IL-17A, Med = 0, SEB = 959.9, SEA = 458.9; IL-22, Med = 110.2, SEB = 3617, SEA = 2177; GM-CSF, Med = 24, SEB = 2697, SEA = 1747. **72 hours**; IFN-γ, Med = 5.8, SEB = 2087, SEA = 2096; IL-2, Med = 6.2, SEB = 1727, SEA = 1480; IL-6, Med = 198.1, SEB = 946.1, SEA = 799.1; TNF-α, Med = 14.2, SEB = 2766, SEA = 1721; IL-17A, Med = 0, SEB = 2205, SEA = 1981; IL-22, Med = 93.71, SEB = 6541, SEA = 5358; GM-CSF, Med = 75.8, SEB = 5705, SEA = 4831. (*) p < 0.05, (**) p < 0.01, (***) p < 0.001, (****) p < 0.0001. NS, not significant.

To verify the functional relevance of our computational simulations, we performed a kinetic analysis of inflammatory cytokine secretion in culture supernatants of highly purified (> 95%) primary CD4^+^ T cells (**Supplementary Figures S1A, B**) stimulated with SEB alone. Consistently with our previous data (27, 43), CD4^+^ T cells isolated from the peripheral blood of healthy donors (HD) expressed CD28 (mean ± SEM = 96.37 ± 2.41) but neither HLA-DR (mean ± SEM = 3.1 ± 0.9) nor B7.1/CD80 (mean ± SEM = 0.98 ± 0.59) nor B7.2/CD86 (mean ± SEM = 1.81 ± 0.88). Stimulation of CD4^+^ T cells with SEB alone induced a strong production of IFN-γ (**Figure 3A**), IL-2 (**Figure 3B**), IL-6 (**Figure 3C**), TNF-α (**Figure 3D**), IL-17A (**Figure 3E**), IL-22 (**Figure 3F**) and GM-CSF (**Figure 3G**) after 48-72 hours. To ascertain that inflammatory cytokine production by CD4^+^ T cells was independent of contaminating accessory cells as found in some samples, we compared SEB-induced inflammatory cytokine production in highly purified CD4^+^ T cells (> 99%, mean = 99.4), expressing very low levels of HLA-DR (mean = 1.8), B7.1/CD80 (mean = 0.5) and B7.2/CD86 (mean = 1.3), and in CD4^+^ T cells with a purity grade between 95-99% (mean = 97.08) (**Supplementary Figure S2**). No significant difference in the amounts of inflammatory cytokines was observed after 72 hours of SEB stimulation in both CD4^+^ T cell populations (**Supplementary Figures S2B-E**). Moreover, stimulation of highly purified CD4^+^ T cells (> 99%) in the presence of increasing numbers (2.5-10%) of murine L-cells (5-3.1/B7) expressing high levels of human HLA-DR1 and B7.1/CD80 (27) did not affect IL-22 and IFN-γ production compared to SEB alone (**Supplementary Figures S2F, G**). A significant increase in TNF-α was observed in T cells stimulated with SEB in the presence of 5% (mean ± SEM = 1390 ± 534) and 10% 5-3.1/B7 cells (mean ± SEM = 1644 ± 282) compared to SEB alone (mean ± SEM = 830 ± 162) (**Supplementary Figure S2H**). Likewise, stimulation of T cells with SEB in the presence of 2.5% (mean ± SEM = 1545 ± 81), 5% (mean ± SEM = 1700 ± 216) and 10% 5-3.1/B7 cells (mean ± SEM = 2056 ± 375) significantly enhanced GM-CSF production compared to SEB alone (mean ± SEM = 1143 ± 305) (**Supplementary Figure S2I**). Similarly to SEB, stimulation of CD4^+^ T cells with SEA elicited comparable amounts of inflammatory cytokines after 48-72 hours of stimulation (**Figures 4A–G**). The analysis of B7.1/CD80, B7.2/CD86 and HLA-DR expression on CD4^+^ T cells after 48-72 hours of stimulation with SEB or SEA revealed no significant changes of B7.1/CD80 (**Supplementary Figure S1C**) or B7.2/CD86 (**Supplementary Figure S1D**). A very low increase of HLA-DR (**Supplementary Figure S1E**) was observed in SEB-stimulated cells (mean ± SEM = 4.72 ± 1.47) compared to unstimulated cells (mean ± SEM = 3.47 ± 1.2).

Altogether these data are consistent with our computational modelling and demonstrate that SEB and SEA are able to directly induce inflammatory cytokine release from T cells independently of MHC class II- and B7-expressing APCs.

### Binding of SEB and SEA to TCR and CD28 elicit inflammatory signals in T cells

**Figure 5:**
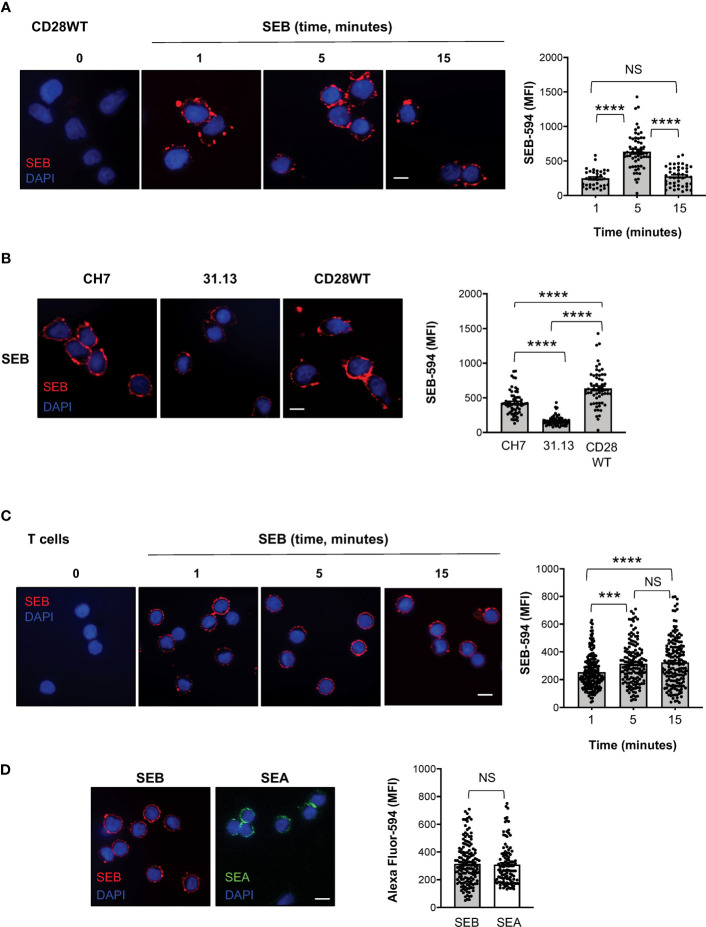
SEB and SEA efficiently bind T cell surface in the absence of APCs. **(A)** Fluorescence microscopy imaging of CH7C17 Jurkat cells expressing human CD28WT incubated for the indicated times with SEB-Alexa Fluor 594 (SEB-594, 1 μg ml^-1^). Nucleus was stained with DAPI (blue). Scale bar = 10 μm. SEB mean fluorescence intensity (MFI) in each single cell was quantified. Bars show the mean ± SEM and statistical significance was calculated by one-way ANOVA. Mean values (SEB-594): 1 minute = 248.5; 5 minutes = 636.7; 15 minutes = 278.2. **(B)** Fluorescence microscopy imaging of CD28-negative CH7C17 (CH7) or CD28WT or TCR-negative 31.13 Jurkat cells incubated for 5 minutes with SEB-594 (1 μg ml^-1^). SEB MFI in each single cell was quantified. Bars show the mean ± SEM and statistical significance was calculated by one-way ANOVA. Mean values (SEB-594): CH7 = 426.9; 31.13 = 170.2; CD28WT = 636.7. **(C, D)** Fluorescence microscopy imaging of CD4+ T cells incubated with for the indicated times with SEB-594 **(C)** or for 5 minutes with SEB-594 or 0.1 μg ml^-1^ SEA-Alexa Fluor 594 **(D)**. SEB and SEA MFI in each single cell was quantified. Bars show the mean ± SEM. Statistical significance was calculated by one-way ANOVA **(C)** or Student’s *t* test (**D**). Mean values (SEB-594): 1 minute = 253.5; 5 minutes = 314.3; 15 minutes = 323.4; SEA-594: 5 minutes = 309.2. Nucleus was stained with DAPI (blue). Scale bar = 10 μm. (***) p < 0.001, (****) p < 0.0001. NS, not significant.

To investigate whether SEB binding to T cells required TCR and/or CD28, we labelled SEB with AlexaFluor-594 and we performed fluorescence microscopy analyses in a CD28-negative CH7C17 Jurkat T cell line expressing TCR Vβ3.1 (37) that specifically interacts with SEB (44), CH7C17 cells reconstituted with human CD28WT (38), and 31.13 Jurkat cells (39) that express CD28 but not TCR/CD3 (**Supplementary Figures S1F, G**). The kinetic analysis performed in CD28WT cells evidenced that SEB efficiently bound to the cell surface with a peak of mean fluorescence intensity (MFI = 636) at 5 minutes (**Figure 5A**). SEB was also able to bind CD28-negative CH7 cells (**Figure 5B**), although with less efficacy as demonstrated by the lower fluorescence intensity (MFI = 427). The requirement of both the TCR and CD28 for optimal SEB binding was also confirmed by the reduced fluorescence intensity (MFI = 170) observed in the TCR-negative 31.13 Jurkat T cell line (**Figure 5B**).

We next analysed the capability of SEB and SEA to directly bind the surface of primary CD4^+^ T cells and to trigger TCR/CD28 inflammatory signals. Consistently with CD28WT cells, the binding of SEB to T cells was detected after 1 minute of incubation and its MFI increased at 5-15 minutes (**Figure 5C**). Similarly, after 5 minutes of incubation, SEA efficiently bound to the surface of T cells with a fluorescence intensity comparable to SEB (**Figure 5D**).

**Figure 6:**
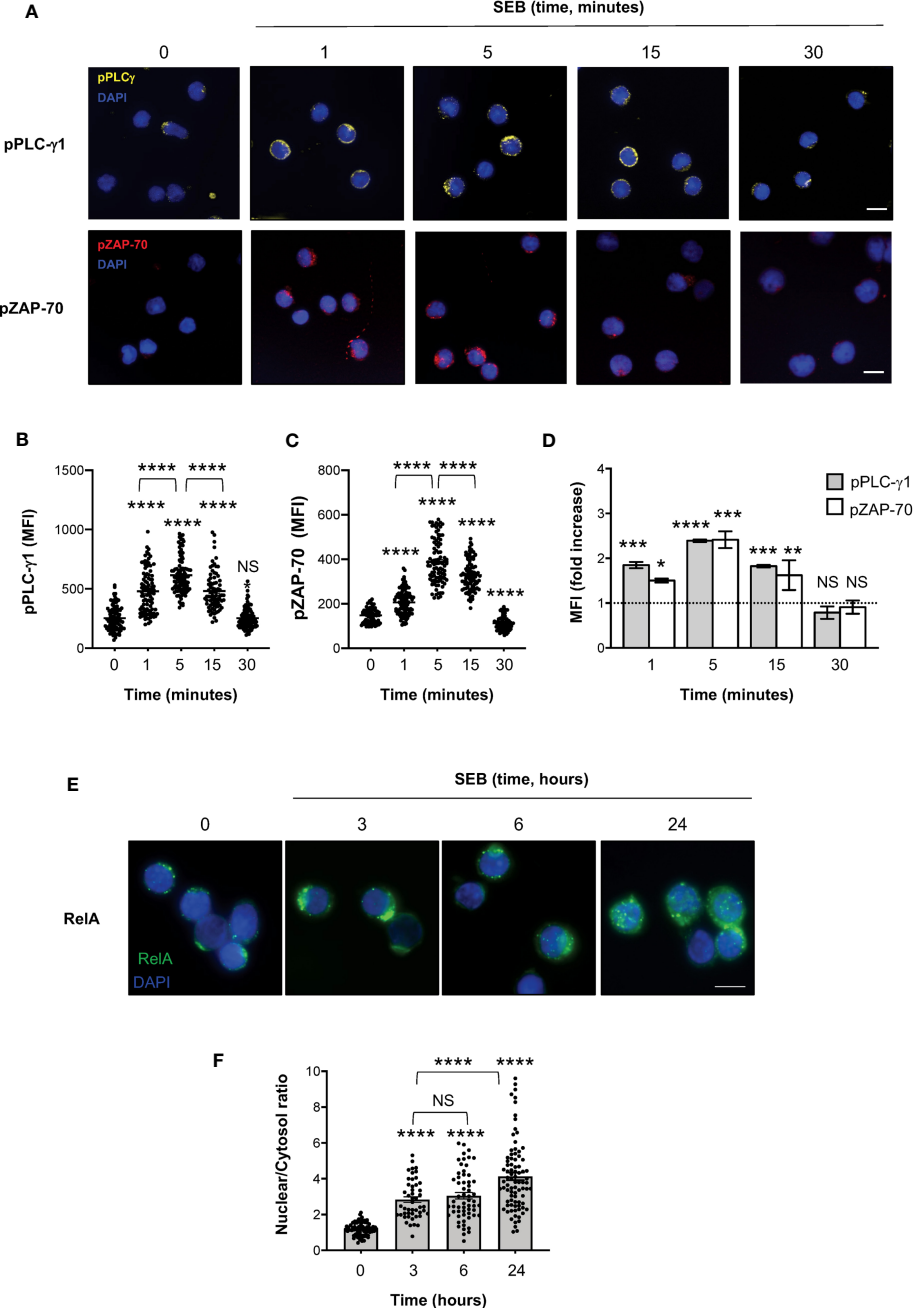
SEB stimulation triggers the activation of both TCR- and CD28-dependent signalling events in CD4^+^ T cells. **(A–D)** Fluorescence microscopy imaging of phosphorylated Y783 PLC-g1 (pPLC-γ1, yellow) or phosphorylated Y319 ZAP-70 (pZAP-70, red) in CD4^+^ T cells unstimulated (0) or stimulated for the indicated times with 1 μg ml^-1^ SEB. MFI of pPLC-γ1 and pZAP-70 in each single cell was quantified. **(B, C)** Bars show the mean ± SEM and statistical significance was calculated by one-way ANOVA. Mean values: pPLC-γ1, 0 = 253.1, 1 minutes = 478.9, 5 minutes = 613.8, 15 minutes = 481.7, 30 minutes = 252.8; pZAP-70, 0 = 148.1, 1 minutes = 206.6, 5 minutes = 388.3, 15 minutes = 328.4, 30 minutes = 112.1. **(D)** MFI fold increase over unstimulated cells was calculated. Data show the mean fold increase ± SEM of three independent experiments. Statistical significance was calculated by one-way ANOVA. Mean values: pPLC-γ1, 1 minutes = 1.84, 5 minutes = 2.39, 15 minutes = 1.82, 30 minutes = 0.78; pZAP-70, 1 minutes = 1.5, 5 minutes = 2.41 15 minutes = 1.62, 30 minutes = 0.9. **(E, F)** Fluorescence microscopy imaging of RelA nuclear translocation in CD4^+^ T cells unstimulated (0) or stimulated for the indicated times with 1 μg ml^-1^ SEB. RelA nuclear and cytoplasmic fractions were quantified in each single cell. Bars show the mean ± SEM of nuclear/cytoplasmic ratio and statistical significance was calculated by one-way ANOVA. Mean values: 0 = 1.19, 3 hours = 2.83, 6 hours = 3.04, 24 hours = 4.13. Nucleus was stained with DAPI (blue). Scale bar = 10 μm. (*) p < 0.05, (**) p < 0.01, (***) p < 0.001, (****) p < 0.0001. NS, not significant.

**Figure 7:**
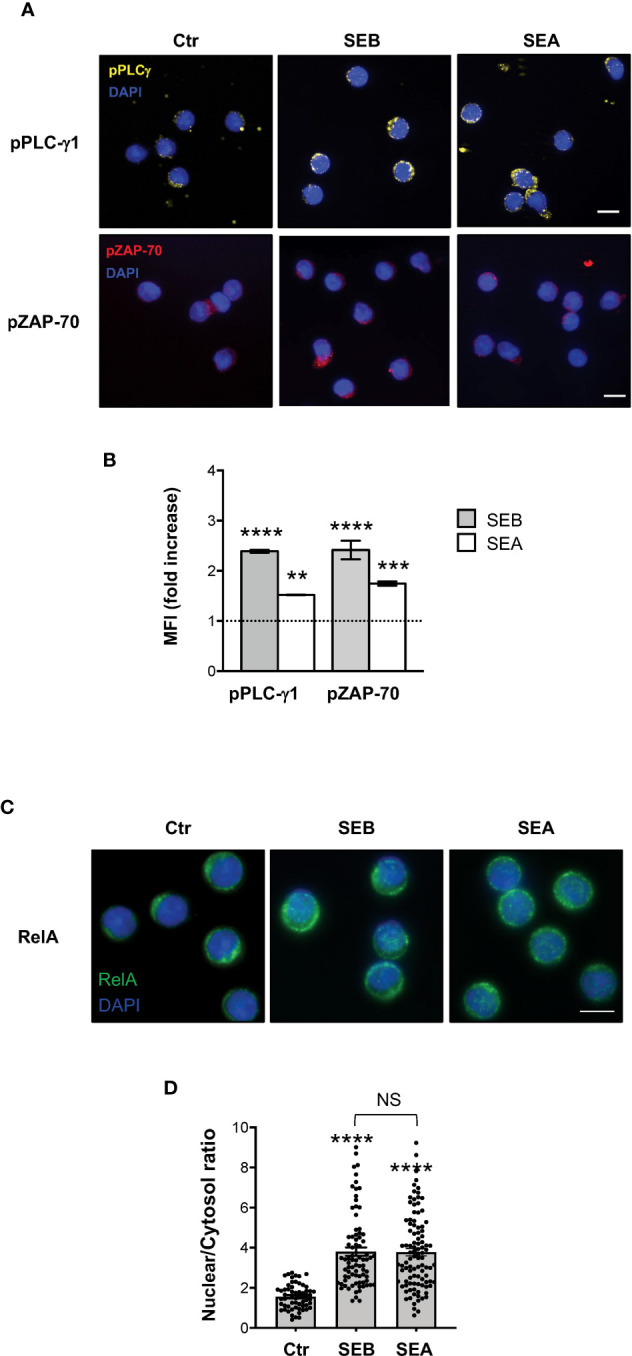
SEB and SEA trigger comparable TCR- and CD28-dependent signalling events in CD4^+^ T cells. **(A, B)** Fluorescence microscopy imaging of pPLC-γ1 (yellow) or pZAP-70 (red) in CD4^+^ T cells unstimulated (0) or stimulated for 5 minutes with 1 μg ml^-1^ SEB or 0.1 μg ml^-1^ SEA. MFI fold increase over unstimulated cells was calculated. Data show the mean fold increase ± SEM of three independent experiments. Statistical significance was calculated by one-way ANOVA. Mean values: pPLC-γ1, SEB = 2.39, SEA = 1.5; pZAP-70, SEB = 2.41, SEA = 1.74. **(C, D)** Fluorescence microscopy imaging of RelA nuclear translocation in CD4^+^ T cells unstimulated (0) or stimulated for 5 minutes with SEB or SEA. RelA nuclear and cytoplasmic fractions were quantified in each single cell. Bars show the mean ± SEM of nuclear/cytoplasmic ratio and statistical significance was calculated by one-way ANOVA. Mean values: 0 = 1.56, SEB = 3.8, SEA = 3.77. Nucleus was stained with DAPI (blue). Scale bar = 10 μm. (*) p < 0.05, (**) p < 0.01, (***) p < 0.001, (****) p < 0.0001. NS, not significant.

To verify whether SEB and SEA binding to the T cell surface induced early and late signalling events activated by both TCR and CD28 co-engagement (24, 45), we analysed the tyrosine phosphorylation of both zeta chain associated protein (ZAP)-70 and phospholipase C (PLC)-γ1 (46) as well as the nuclear translocation of RelA/nuclear factor-kappa B (NF-κB) subunit (47–49). In T cells, SEB induced a significant and sustained increase of phosphorylation of both Y783 in PLC-γ1 (**Figures 6A, B**) and Y319 in ZAP-70 (**Figures 6A, C**), whose MFI began to increase within 1 minute after stimulation (fold increase: pPLC-γ1 = 1.8; pZAP-70 = 1.5), reached a peak at 5 minutes (fold increase: pPLC-γ1 and pZAP-70 = 2.4), started to decline after 15 minutes (fold increase: pPLC-γ1 = 1.8; pZAP-70 = 1.6) and returned to a basal level after 30 minutes (**Figure 6D**, fold increase: pPLC-γ1 = 0.7; pZAP-70 = 0.9). Fluorescence microscopy analysis of the nuclear localization of RelA/NF-κB (50) revealed that SEB triggered a significant and sustained increase (over 24 hours) of RelA/NF-κB nuclear translocation (**Figures 6E, F**). Similar data were obtained in T cells following stimulation with SEA that elicited a significant increase of both Zap-70 and PLC-γ1 tyrosine phosphorylation (**Figures 7A, B**) as well as of RelA/NF-κB nuclear translocation (**Figures 7C, D**).

Taken together these findings show that SEB and SEA might directly bind to both TCR and CD28 and activate the early and late signalling events associated with inflammatory cytokines.

### SEB and SEA colocalization with the TCR and CD28 in primary CD4^+^ T cells

**Figure 8:**
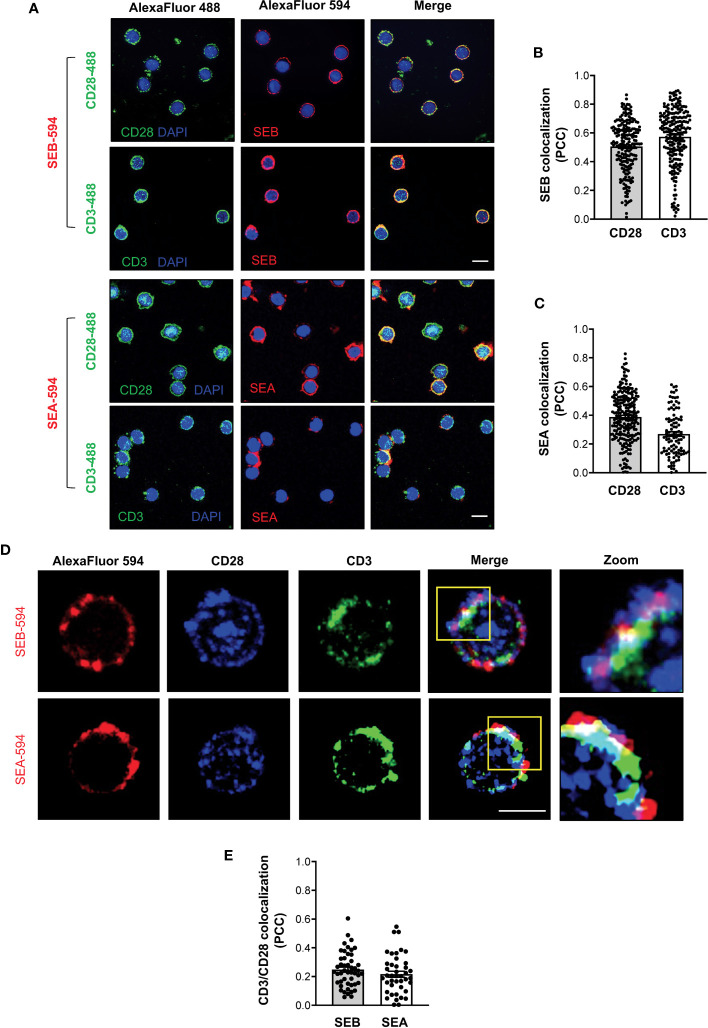
SEB and SEA colocalize with the TCR and CD28 in CD4^+^ T cells. **(A-C)** Primary CD4^+^ T cells were incubated for 5 minutes with 1 μg ml^-1^ SEB-AlexaFluor 594 or 0.1 μg ml^-1^ SEA-AlexaFluor 594 for 5 minutes. After fixing and permeabilization CD28 (upper panel) or CD3 (lower panel) were stained with anti-CD28 or anti-CD3 followed by AlexaFluor 488-coniugated secondary Abs and analysed by confocal microscopy. Nucleus was stained with DAPI (blue). Scale bar = 10 μm. The Pearson correlation coefficient (PCC) was calculated by analysing the degree of colocalization between SEB-594 **(B)** or SEA-594 **(C)** and CD28-488 or CD3-488. Bars show the mean ± SEM of PCC values. Mean PCC: SEA, CD3 = 0.27, CD28 = 0.39; SEB, CD3 = 0.57, CD28 = 0.5. **(D)** Confocal microscopy imaging of primary CD4^+^ T cells incubated for 5 minutes with SEB-AlexaFluor 594 (upper panel) or SEA-AlexaFluor 594 (lower panel) and stained with rabbit anti-human-CD28 followed by anti-rabbit-AlexaFluor 647, and mouse anti-human-CD3 followed by anti-mouse-AlexaFluor 488. **(E)** PCC was calculated by analysing the degree of colocalization of CD28-647 and CD3-488 fluorescence in SEB-594- or SEA-594-stained cells. Bars show the mean ± SEM of PCC values. Mean PCC: SEA, CD3/CD28 = 0.21; SEB, CD3/CD28 = 0.24. Scale bar = 10 μm.

To demonstrate the capability of both SEB and SEA to bind to the TCR and CD28 in a bivalent manner, we evaluated the colocalization of either SEB or SEA with the TCR and CD28 in primary CD4^+^ T cells. Confocal microscopy analysis evidenced that soon after 5 minutes of stimulation, both SEB and SEA (**Figure 8A**) efficiently colocalize with either CD3 or CD28, as demonstrated by the values of the Pearson correlation coefficient (PCC) (**Figures 8B, C**). Moreover, in stimulated CD4^+^ T cells (**Figure 8D**), we also found a substantial colocalization of both CD28 and CD3 with either SEB or SEA (**Figure 8E**), as evidenced by PCC values (SEB, mean PCC = 0.25; SEA, mean PCC = 0.22), suggesting a bivalent binding of either SEB or SEA to the TCR and CD28.

Altogether these data support our computational and functional data on the capability of staphylococcal SAgs to promote the activation of pro-inflammatory signals by simultaneously engaging the TCR and CD28 even in the absence of APCs expressing MHC class II and B7 molecules.

## Discussion

For several decades, T-cell activation by bacterial SAgs was believed to require only the TCR and MHC class II molecules (12). However, the discovery of CD28 and B7 molecules as novel co-ligands of staphylococcal SAgs (20, 21, 23, 26) together with our recent data showing that the binding of SEB to B7 molecules on APCs and to CD28 on T cells leads to a productive engagement of the TCR even in the absence of MHC class II, changed this classical view (27). Here, we show that SEB- and SEA-mediated activation of inflammatory signals in T cells also can occur in the absence of MHC class II- and B7-expressing APCs.

Starting from the available experimental data on the complex between SEB, the TCR and MHC class II (19), we initially rebuilt a tentative model of the whole SEB-MHCII-TCR-CD3γϵ-CD3δϵ-CD3ζζ signalling hexamer by exploiting the recently released cryo-EM structure (32). This first model permitted us to pinpoint the orientation of SEB relative to the lipid bilayer and the surface regions left for interaction with CD28. Then, using the crystal structures of CD28 (29, 30) and a spatial-restrained protein-protein docking approach based on previous experimental data (18, 19, 21), we obtained a final complex that permitted us to analyse, from a structural point of view, the interaction network of SEB with its ligands (**Figure 1A**; **Supplementary Movie S1**). The same procedure was adopted for the highly similar SEA (**Figure 1B**; **Supplementary Movie S2**), and the two obtained models suggested that most of the interaction surfaces of SEA and SEB were occupied by contacts with the TCR and CD28 (**Figure 2**). These data suggest that the co-engagement of the TCR and CD28 by staphylococcal SAgs might suffice for the activation of inflammatory signals. Indeed, we demonstrate here that both SEA and SEB induce a strong production of inflammatory cytokines in highly purified CD4^+^ T cells in the absence of APCs (**Figures 3**, **4**; **Supplementary Figure S2**). These data support the capability of SEA and SEB to cooperatively interact with both the TCR and CD28 eliciting inflammatory signals.

Efficient T-cell activation and inflammatory cytokine production require both TCR and CD28 signals (48, 51). Generally, both signals are initiated at the immunological synapse (IS) following the engagement of the TCR and CD28 by peptide-MHC complexes and B7 molecules on APCs, respectively (52). Similarly to the IS, in the superantigen synapse, the engagement of CD28 and B7 by staphylococcal SAgs together with the TCR triggers both TCR- and CD28-dependent signals, thus leading to optimal T cell activation (24, 51, 53) and inflammatory responses (20, 21, 23, 26), even in the absence of MHC class II co-engagement (27). However, primary CD4^+^ T cells may generate mechanical forces through the TCR and CD28 when stimulated by high affinity ligands also in the absence of cell-cell interaction, thus leading to productive TCR- and CD28-activating signals (54, 55). Here we show that staphylococcal SAgs efficiently bind to the surface of TCR^+^CD28^+^ Jurkat cells and primary CD4^+^ T cells (**Figure 5**). Notably, the binding of SAgs was maximal when both TCR and CD28 were expressed, as evidenced by the reduction of SEB binding observed in TCR^-^CD28+ 31.13 or TCR^+^CD28^-^ Jurkat cells compared to TCR^+^ CD28WT cells (**Figure 5B**). Although the binding affinities of SEA and SEB for the TCR and CD28 are quite similar (20, 56, 57), the binding of both SAgs to the cell surface increased when both the TCR and CD28 were co-expressed (**Figure 5B**). These data are consistent with the findings that TCR stimulation activates an inside-out signalling that induces the formation of a more stable CD28 homodimer interface (58–60), likely favouring an optimal binding of staphylococcal SAgs as well as signalling (20). For instance, the mitogenicity of CD28 ligands depends on the epitope location (29) as evidenced by ability of superagonistic Abs to induce CD28 clustering and signalling by binding in a bivalent way a loop close to the homodimer interface (61). Likewise, by simultaneously binding the TCR and CD28, SEB and SEA might act in a superagonistic way triggering optimal activating signals.

It is well known that the TCR and CD28 cooperate to activate the early and late signalling events regulating cytokine production (48, 51, 62). The phosphorylation of several tyrosine residues within ZAP-70, including Y319 (63, 64), is one of the earliest signalling events activated following TCR engagement and is required for ZAP-70 full activation (46). Active ZAP-70 in turn phosphorylates several critical signalling mediators, including PLC-γ1 on Y783 (65, 66), thus leading to the activation of both Ca^2+^ and PKC signalling pathways (67–69). TCR and CD28 signalling pathways are strictly interdependent through a dual-positive-feedback loop (70). For instance, TCR-dependent ZAP-70 recruitment and activation (71) elicits a PLC-γ1-dependent Ca^2+^ response that in turn induce a conformational change in the CD28 cytoplasmic domain facilitating CD28 signalling (70). On the other hand, CD28 enhances and sustains early TCR signalling (24, 72–74) by favouring the actin cytoskeleton events required for the recruitment of critical signalling mediators to both the TCR and CD28 (38, 41, 75–77). Consistently, we found that SEB and SEA binding to the TCR and CD28 triggers a sustained activation of both ZAP-70 and PLC-γ1 (**Figures 6A-D**; **Figures 7A, B**). Moreover, both SEB and SEA also induced the nuclear translocation of RelA/NF-κB (**Figures 6E, F**; **Figures 7C, D**), an important transcription factor that is involved in inflammatory cytokine gene expression and is mainly regulated by the CD28 signalling axis (41, 48, 78–81).

Finally, our data on the colocalization of both SEB and SEA with either CD3 or CD28 together with the colocalization of both CD3 and CD28 with SEB or SEA on the surface of primary CD4^+^ T-cells (**Figure 8**), suggest that staphylococcal SAgs might function as bivalent stimulators of the TCR and CD28. For instance, by binding simultaneously to the TCR and CD28, staphylococcal SAgs may induce the dimerization and/or conformational changes required for triggering the early tyrosine phosphorylation events, which in turn will promote the recruitment of important signalling mediators and the activation of downstream inflammatory cascades, as occur for receptor tyrosine kinases (82, 83).

Altogether, our data reveal a novel model of T-cell activation by bacterial SAgs, which share the ability to bind both TCR and CD28/B7 molecules, exemplified by staphylococcal and streptococcal toxins (23, 26), and provides new insight for the design of novel SAg antagonists able to interfere with the formation of SAg-TCR-CD28 complex, thus inhibiting massive inflammatory signals.

## Data availability statement

The raw data supporting the conclusions of this article will be made available by the authors, without undue reservation.

## Ethics statement

The studies involving human participants were reviewed and approved by Ethical Committee of the Policlinico Umberto I (Sapienza University, Rome, Italy). The patients/participants provided their written informed consent to participate in this study.

## Author contributions

MK performed most of the experiments, analysed the data, interpreted the results and helped in writing the manuscript. CA performed part of the experiments and data analyses. FS performed part the experiments and contributed to discussions and editing the manuscript. SC contributed with technical support. MF contributed to discussions and editing the manuscript. AP performed the computational analyses and contributed to discussions, writing and editing the manuscript. RK contributed to discussions and editing the manuscript. LT designed the study, coordinated the work and wrote the manuscript. All authors contributed to the article and approved the submitted version.

## In memoriam

This paper is dedicated to the memory of our friend and colleague Silvana Caristi who prematurely died in 2022.

## Supplementary material

Supplementary Movie S1 Predicted model of the TCR–SEB–CD28 complex. Cartoon representation with transparent surfaces of the overall structure. TCR is depicted in cyan, SEB in brown and CD28 in green. The lipid bilayer is shown as reference. Shadowed surfaces ofMHC class II (MHCII), B7.2/CD86 (B7) and the CD3γε-CD3δε-CD3ζζ signaling hexamers are also shown as reference.

Supplementary Movie S2 Predicted model of the TCR–SEA–CD28 complex. Crystal structures of SEA is depicted in pink, mixed model/crystal structure of TCR in cyan and CD28 in green. The position of SEA and TCR is taken by the corresponding crystal structure of the complex(PDB: 5FK9).
