# Corrigendum: Bivalent binding of staphylococcal superantigens to the TCR and CD28 triggers inflammatory signals independently of antigen presenting cells

**DOI:** 10.3389/fimmu.2023.1273921

**Published:** 2023-08-15

**Authors:** Martina Kunkl, Carola Amormino, Francesco Spallotta, Silvana Caristi, Maria Teresa Fiorillo, Alessandro Paiardini, Raymond Kaempfer, Loretta Tuosto

**Affiliations:** ^1^ Department of Biology and Biotechnology “Charles Darwin”, Sapienza University, Rome, Italy; ^2^ Laboratory affiliated to Istituto Pasteur Italia-Fondazione Cenci Bolognetti, Sapienza University, Rome, Italy; ^3^ Department of Biochemical Sciences “A. Rossi Fanelli”, Sapienza University of Rome, Rome, Italy; ^4^ Department of Biochemistry and Molecular Biology, The Institute for Medical Research Israel-Canada, The Hebrew University-Hadassah Medical School, Jerusalem, Israel

**Keywords:** staphylococcal superantigens, T cells, TCR (T cell receptor), CD28, inflammation

In the published article, there was an error in **Figure 3** as published. **Figure 3** was substituted with Figure 4, which was duplicated. The corrected **Figure 3** and its caption appear below.

**Figure 3:**
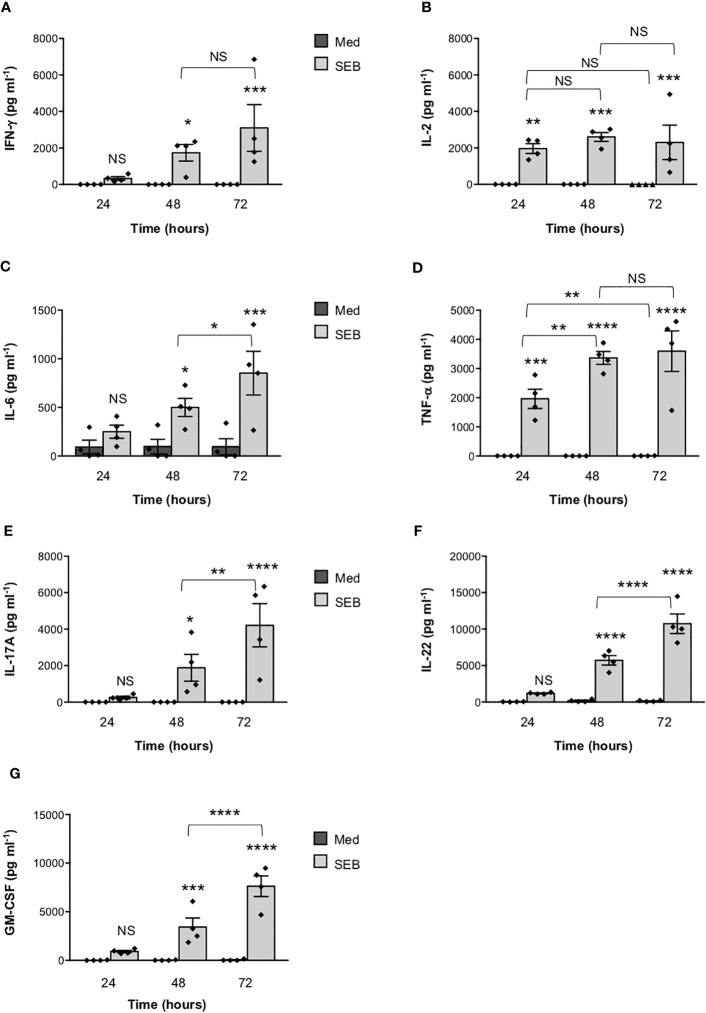
Stimulation of CD4+ T cells by SEB induces the secretion of inflammatory cytokines in the absence of MHC class II- and B7-expressing APCs. **(A–G)** Human CD4+ T cells isolated by the peripheral blood of healthy donors (HD) were unstimulated (Med) or stimulated for different times with 1 μg ml-1 SEB. IFN-γ **(A)**, IL-2 **(B)**, IL-6 **(C)**, TNF-α **(D)**, IL-17A **(E)**, IL-22 **(F)** and GM-CSF **(G)** levels in culture supernatant were measured by ELISA. Data show the mean ± SEM of different HD (n = 4). Statistical significance was calculated by One-way ANOVA. Means values (pg ml-1): 24 hours; IFN-γ, Med = 0, SEB = 328.7; IL-2, Med = 0, SEB = 1966; IL-6, Med = 92.2; SEB = 249; TNF-α, Med = 0, SEB = 1959; IL-17A, Med = 0, SEB = 251.7; IL-22, Med = 37.4, SEB = 1197; GM-CSF, Med = 19.1, SEB = 916.5. 48 hours; IFN-γ, Med = 0, SEB = 1738; IL-2, Med = 0, SEB = 2600; IL-6, Med = 96.7, SEB = 498.8; TNF-α, Med = 0, SEB = 3361; IL-17A, Med = 0, SEB = 1884; IL-22, Med = 178.6, SEB = 5726; GM-CSF, Med = 22,3, SEB = 3426. 72 hours; IFN-γ, Med = 0, SEB = 3099; IL-2, Med = 0.9, SEB = 2303; IL-6, Med = 95, SEB = 851.5; TNF-α, Med = 2.6, SEB = 3591; IL-17A, Med = 0, SEB = 4211; IL-22, Med = 140.9, SEB = 10737; GM-CSF, Med = 42.3, SEB = 7633. (*) p < 0.05, (**) p < 0.01, (***) p < 0.001, (****) p < 0.0001. NS, not significant.

The authors apologize for this error and state that this does not change the scientific conclusions of the article in any way. The original article has been updated.

