# Supplementary material for: Bivalent binding of staphylococcal superantigens to the TCR and CD28 triggers inflammatory signals independently of antigen presenting cells
Source: Front Immunol. 2023 May 3;14:1170821. doi: 10.3389/fimmu.2023.1170821 (PMC10189049; doi:10.3389/fimmu.2023.1170821)
Supplement: Supplementary file 3 [file DataSheet_1.pdf]

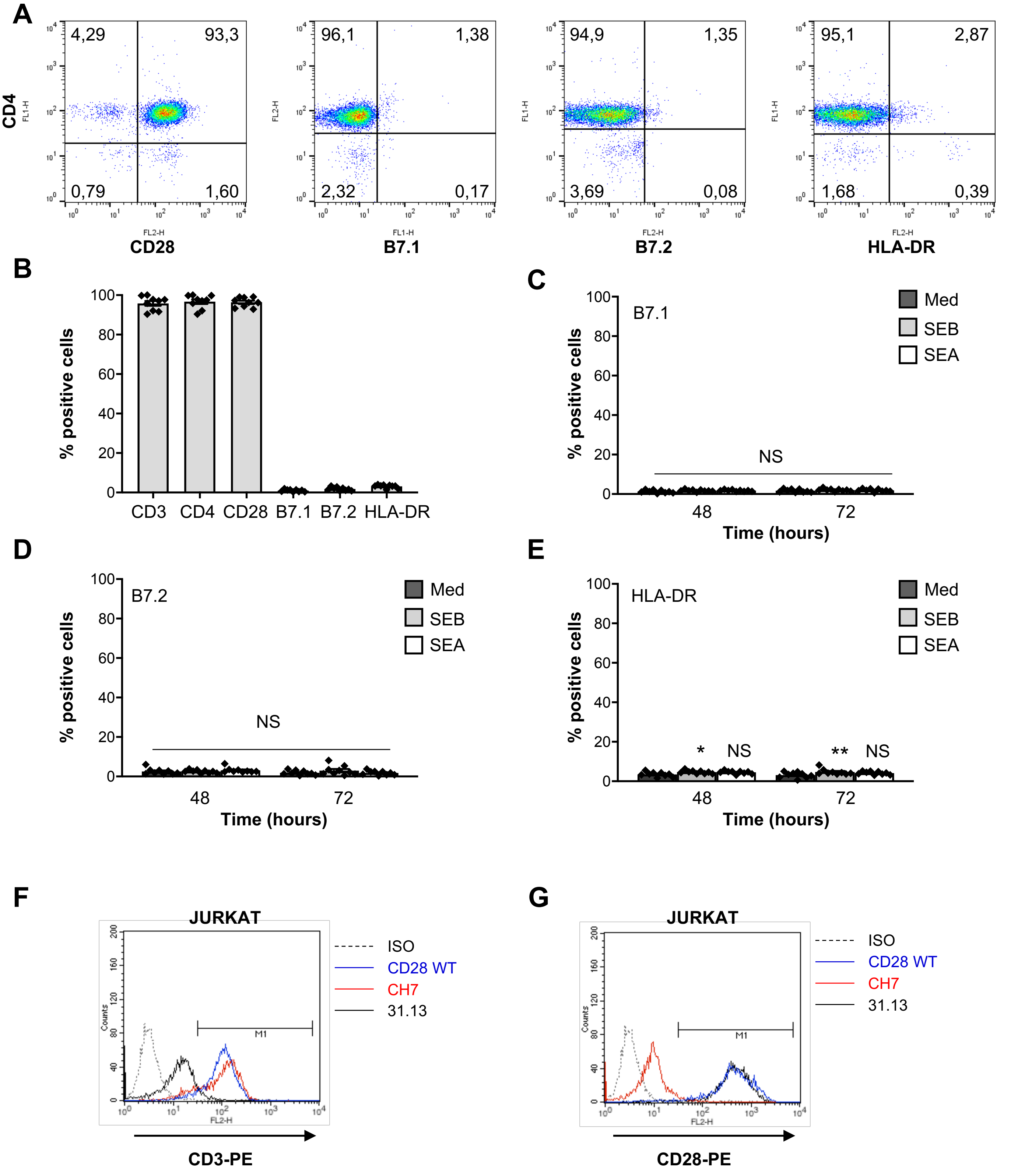

Supplementary Figure S1. Phenotypic analysis of human CD4⁺ T cells and Jurkat T cell lines. (**A**) Representative FACS analysis of human CD4⁺ T cells stained with anti-CD4-FITC plus anti-CD28-PE or anti-B7.2-PE or anti-HLA-DR-PE or anti-CD4-PE plus anti-B7.2-FITC. (**B-E**) The percentage of human CD4+ T cells from HD (n = 9) expressing CD3, CD28 B7.1, B7.2 and HLA-DR, were calculated after purification and following stimulation with SEB, or SEA, for 48h and 72h. (**F**, **G**) FACS analysis of CD3 and CD28 in CD28WT, CH7 and 31.13 cells. Iso = isotype control Abs.
