# Supplementary material for: Bivalent binding of staphylococcal superantigens to the TCR and CD28 triggers inflammatory signals independently of antigen presenting cells
Source: Front Immunol. 2023 May 3;14:1170821. doi: 10.3389/fimmu.2023.1170821 (PMC10189049; doi:10.3389/fimmu.2023.1170821)
Supplement: Supplementary file 4 [file DataSheet_2.pdf]

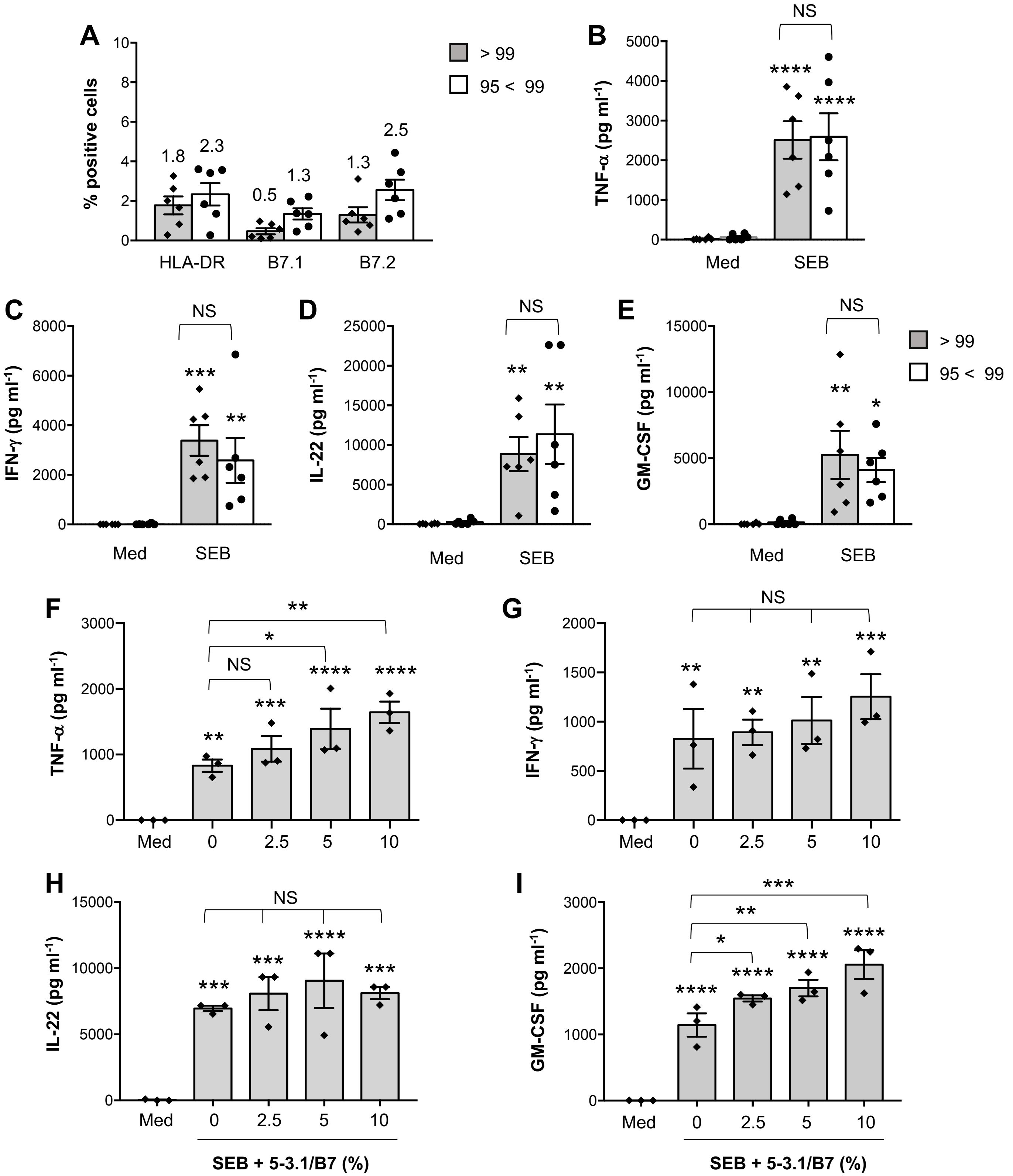


**Supplementary Figure S2. Inflammatory cytokine production in highly purified human CD4+ T cells.** (**A**) Percentage of B7.1, B7.2 and HLA-DR expression (values indicate the mean %) in highly purified CD4+ T cells (> 99, mean = 99.4) or CD4+ T cells with a purity grade between 95-99% (mean = 97.08). (**B-I**) Inflammatory cytokine production in culture supernatants of highly purified (> 99) or 95 < 99 pure CD4+ T cells unstimulated (Med) or stimulated with 1 μg $ml^{-1}$ SEB for 72 hours (**B-E**) or highly purified CD4+ T cells (> 99) stimulated for 72 hours with SEB in the absence or presence of the indicated percentage of 5-3.1/B7 cells (**F-I**). Data show the mean ± SEM and statistical significance was calculated by one-way ANOVA. (*) $p < 0.05$, (**) $p < 0.01$, (***) $p < 0.001$, (****) $p < 0.0001$, NS = not significant.
